# Severity Classification Using Dynamic Time Warping–Based Voice Biomarkers for Patients With COVID-19: Feasibility Cross-Sectional Study

**DOI:** 10.2196/50924

**Published:** 2023-11-06

**Authors:** Teruhisa Watase, Yasuhiro Omiya, Shinichi Tokuno

**Affiliations:** 1 Gradutate School of Health Innovation Kanagawa University of Human Service Kawasaki, Kanagawa Japan; 2 Department of Bioengineering Graduate School of Engineering The University of Tokyo Tokyo Japan

**Keywords:** voice biomarker, dynamic time warping, COVID-19, smartphone, severity classification, biomarker, feasibility study, illness, monitoring, respiratory disease, accuracy, logistic model, tool, model

## Abstract

**Background:**

In Japan, individuals with mild COVID-19 illness previously required to be monitored in designated areas and were hospitalized only if their condition worsened to moderate illness or worse. Daily monitoring using a pulse oximeter was a crucial indicator for hospitalization. However, a drastic increase in the number of patients resulted in a shortage of pulse oximeters for monitoring. Therefore, an alternative and cost-effective method for monitoring patients with mild illness was required. Previous studies have shown that voice biomarkers for Parkinson disease or Alzheimer disease are useful for classifying or monitoring symptoms; thus, we tried to adapt voice biomarkers for classifying the severity of COVID-19 using a dynamic time warping (DTW) algorithm where voice wavelets can be treated as 2D features; the differences between wavelet features are calculated as scores.

**Objective:**

This feasibility study aimed to test whether DTW-based indices can generate voice biomarkers for a binary classification model using COVID-19 patients’ voices to distinguish moderate illness from mild illness at a significant level.

**Methods:**

We conducted a cross-sectional study using voice samples of COVID-19 patients. Three kinds of long vowels were processed into 10-cycle waveforms with standardized power and time axes. The DTW-based indices were generated by all pairs of waveforms and tested with the Mann-Whitney *U* test (α<.01) and verified with a linear discrimination analysis and confusion matrix to determine which indices were better for binary classification of disease severity. A binary classification model was generated based on a generalized linear model (GLM) using the most promising indices as predictors. The receiver operating characteristic curve/area under the curve (ROC/AUC) validated the model performance, and the confusion matrix calculated the model accuracy.

**Results:**

Participants in this study (n=295) were infected with COVID-19 between June 2021 and March 2022, were aged 20 years or older, and recuperated in Kanagawa prefecture. Voice samples (n=110) were selected from the participants’ attribution matrix based on age group, sex, time of infection, and whether they had mild illness (n=61) or moderate illness (n=49). The DTW-based variance indices were found to be significant (*P*<.001, except for 1 of 6 indices), with a balanced accuracy in the range between 79% and 88.6% for the /a/, /e/, and /u/ vowel sounds. The GLM achieved a high balance accuracy of 86.3% (for /a/), 80.2% (for /e/), and 88% (for /u/) and ROC/AUC of 94.8% (95% CI 90.6%-94.8%) for /a/, 86.5% (95% CI 79.8%-86.5%) for /e/, and 95.6% (95% CI 92.1%-95.6%) for /u/.

**Conclusions:**

The proposed model can be a voice biomarker for an alternative and cost-effective method of monitoring the progress of COVID-19 patients in care.

## Introduction

### Background

COVID-19 originated in Wuhan, China, in December 2019 and turned into a worldwide pandemic. As of December 2022, the number of people infected with this disease reached approximately 650 million, of whom more than 6.64 million had lost their lives. Although the number of new infections appeared to have abated in the spring of 2023, the past explosion of infections strained medical care systems in several countries. To cope with this pressure, these countries have changed their responses toward infected patients based on the severity of their illness. In Japan, as defined in Table 1, which shows the Ministry of Health, Labour and Welfare guidelines on the severity of COVID-19 [1], responses were divided into 4 categories of severity, ranging from mild to serious illness. Mild illness is defined as “an oxygen saturation of 96% or more, or a clinical condition of no respiratory symptoms or coughing without shortness of breath (SoB) but no evidence of pneumonia in either case,” and moderate illness I is defined as “an oxygen saturation of greater than 93% but less than 96%, or a clinical condition of shortness of breath or pneumonia.” Moderate illness II is defined as “oxygen saturation of 93% or less, or oxygen administration is required.” The target population for this study was individuals who were recovering at home or in recuperation facilities. Therefore, they were theoretically patients with mild illness. Still, due to worsening conditions or shortcomings of medical services, this population included patients with moderate illness I who should have been treated in a hospital. Therefore, accurately classifying these 2 adjacent severity categories (mild illness and moderate illness I) is essential in determining appropriate measures, such as early hospitalization, by detecting worsening conditions in patients with mild illness.

**Table 1:** Definitions of the severity of COVID-19 infections.

Severity	Oxygen saturation, %	Clinical condition
Mild illness	≥96	Absence of respiratory symptoms or presence of coughing without shortness of breath, but no evidence of pneumonia in either case
Moderate illness I	93-96	Shortness of breath and pneumonia are evident
Moderate illness II	≤93	Oxygen administration is required
Serious illness	N/A	Admission to intensive care unit or requirement of a ventilator

Oxygen saturation (SpO_2_) measurements using a pulse oximeter were crucial for assessing the severity of illness. Daily measurements of SpO_2_ and body temperature, along with the assessment of physical conditions, were essential for monitoring disease progression from mild illness to moderate illness I over approximately 1 week or more during the recuperation period. However, the explosive increase in the number of COVID-19 patients made it difficult to distribute pulse oximeters to all patients with mild illness, especially young patients who were forced to recuperate at their homes rather than in health care facilities. This unexpected shortage of pulse oximeters has motivated us to devise alternative and cost-effective ways to monitor for worsening medical condition in persons exhibiting mild illness.

### Voice Biomarkers

Previous research on Parkinson disease, Alzheimer disease, depression, and other psychiatric disorders such as stress [2-7] has shown that voice biomarkers can be leveraged to noninvasively and cost-effectively identify the presence or absence of diseases, classify symptoms, and monitor conditions. Voice biomarkers could also be an alternative method to detect changes in disease severity from mild illness to moderate illness I in COVID-19, which is a respiratory disease and has been reported to cause acoustic changes in the voice due to inflammation of the pharynx in the vocal tract, vocal cords, or both, as well as a lower expiratory volume due to pneumonia [8]. Moreover, significant differences in jitter (fluctuation of the fundamental frequency on the time axis), shimmer (fluctuation of the amplitude on the power axis), and harmonic-to-noise ratio (HNR) were reported between healthy subjects and those with COVID-19 [8-11]. There are also reports that COVID-19 can be detected from acoustic data obtained from a patient’s cough [12].

### Dynamic Time Warping

**Figure 1:**
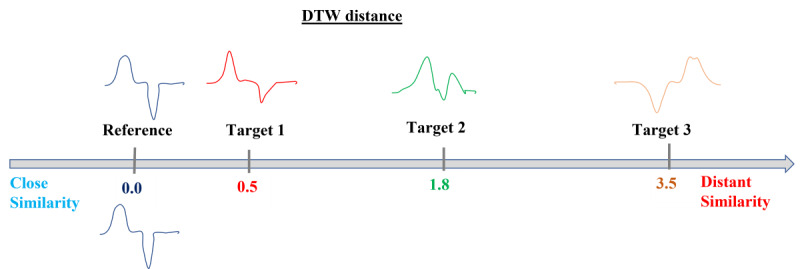
Illustration of the DTW distance, with 3 target example waves compared to a reference wave. DTW: dynamic time warping.

Dynamic time warping (DTW) is an effective algorithm for measuring the similarity between 2 patterns. The DTW distance, which is a computational result obtained by the DTW algorithm using 2 waveform features, progressively approaches zero as the features become more similar, whereas it increases as the features become less similar (Figure 1).

This metric has been widely used since the 1980s in fields such as motion recognition, speech recognition, and time-series data analysis [13-15]. For example, it was reported that DTW could differentiate between healthy people and people with walking disabilities with high accuracy by processing differences in the gait patterns acquired by accelerometer sensors on smartphones [13]. The effectiveness of an automated scoring system applied in conjunction with the DTW algorithm for evaluating the progress of speech audiometric rehabilitation was reported to be similar to that of conventional manual scoring methods [14]. It has also been reported that complementing the mel-frequency cepstral coefficient (MFCC) algorithm with the DTW algorithm improved voice recognition performance. The DTW algorithm has been introduced as a feature-matching technique for voice recognition [15]. The feature-matching performance of the DTW algorithm (ie, the scoring method for 2D feature similarity) may function effectively for the desired classification of long vowel samples.

### Goal of This Study

This feasibility study aimed to test whether DTW-based voice biomarkers can be used to achieve a binary classification of mild illness and moderate illness I for COVID-19 at a significant level.

## Methods

### Study Design

We conducted a cross-sectional study using the voice samples of COVID-19 patients.

### Participants

This study recruited participants through a brochure that was distributed exclusively to COVID-19 patients who were aged 20 years and older, were positive for SARS-CoV-2 in PCR testing, and recuperated at designated facilities or at home in Kanagawa prefecture, Japan, between June 2021 and March 2022. Patients who consented to the study’s objectives were requested to register for participation using the QR code on the brochure through their smartphones. The participants were asked to provide their daily vital signs data, including voice recordings during the recuperation period, but were also given the option to withdraw from the study (opt out) at any time of their own accord. A ¥1000 (US $6.68) Amazon gift card was given to participants as compensation. Because this study only included patients with mild illness or moderate illness I, who did not require hospitalization, patients with moderate illness II were not included. The participants were divided into 2 groups, the mild group and moderate I group, according to the definitions given in Table 1.

### Data Collection

Those who agreed to participate in the study were asked to install a smartphone app and enter their basic information, vital signs data (temperature and SpO_2_), symptom scores, and voice recordings on the first day of recuperation. From the second day onward, the participants were required to enter their vital signs data, symptom scores, and voice recordings daily until the last day of recuperation. Voice data were stored together with text data indicating the symptoms and vital signs on a dedicated server with high security. The voice recordings were in the WAV format with a sampling rate of 48 kHz and a bit depth of 16 bits using the 3 long vowels /a/, /e/, and /u/. Participants were asked to explain their reasoning if they wished to withdraw from the study. Table 2 shows the timing and data entry items of the participants.

**Table 2:** Timing and items for the input data and information from participants.

Timing and items		Description
**Baseline**		
	Orientation	The researchers explained the purpose of the study and obtained participant consent
	Basic information	Sex Age Symptom onset date Diagnosis confirmation date Treatment start date
**On a daily basis during recuperation**		
	Vital sign data	Body temperature Blood oxygen saturation
	Questionnaire	Change in symptoms Symptomatic or not Respiratory distress Taste or olfactory disorders Cough or sputum Chest pain Runny nose or nasal congestion Sore throat Nausea or vomiting Diarrhea Appetite Fatigue Headache Joint pain Rash Red eyes
	Voice recording	Three long vowels: /a/, /e/, /u/
**Dropout during recuperation**		
	Dropout	Confirm the reason for dropping out from the research

### Waveform Sample Cutout and Standardization

**Figure 2:**
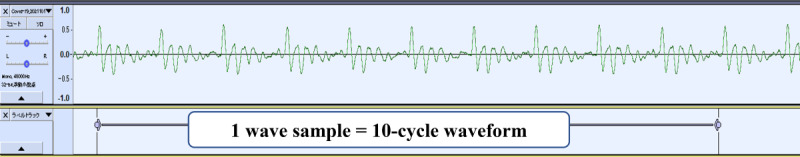
Screenshot showing 10-cycle waveform data extracted for each date from each patient’s voice recording of vowels.

To calculate the DTW distance, a 10-cycle waveform sample was extracted for each date from the participants’ long-vowel recordings in the WAV format using Audacity (version 3.1.3; Audacity Team) at a sampling rate of 48 kHz (Figure 2). Then, standardization was achieved along the power axis within the range of –1 to +1 as the maximum amplitude, and the time axis involved 1000 data points that were multiplied by 1/48,000 seconds, considering the length of a 10-cycle waveform. To read and standardize the WAV data, R (version 4.4.2; R Core Team) with the *tuneR* package (version 1.4.0) was used.

### Calculation of the DTW Distance for 2 Groups

After standardizing the power and time of the 110 waveform samples, the DTW distance was calculated for each sample paired with those of the remaining 109 samples. The DTW distances that were obtained were divided into 2 categories based on 2 kinds of labels for the 109 waveform samples. Therefore, each sample was assigned 2 variables for DTW distance. In the mild group, these were 61 or 60 DTW distances, and in the moderate I group, these were 48 or 49 DTW distances. The average and variance of the DTW distances were calculated for each group. For the mild group, the average index (ie, the mild-group filtering [MiF] average) and variance index (ie, MiF variance) of the DTW distance were obtained, whereas for the moderate I group, the average index (ie, moderate-group filtering [MoF] average) and variance index (MoF variance) of the DTW distance were obtained. These 4 indices were obtained from a single waveform sample. The indices for the 3 vowels, /a/, /e/, and /u/, were prepared and are represented as shown in Table 3. Thus, 12 indices were used in the subsequent analyses. The average values and variances of the DTW distances for the 2 groups were statistically investigated to determine whether they exhibited significant values for the 2-group classification scheme.

**Table 3:** Twelve indices generated from 3 vowels and 4 indices.

Indices	Vowels		
	/a/	/e/	/u/
MiF^a^ average	/a/-MiF average	/e/-MiF average	/u/-MiF average
MoF^b^ average	/a/-MoF average	/e/-MoF average	/u/-MoF average
MiF variance	/a/-MiF variance	/e/-MiF variance	/u/-MiF variance
MoF variance	/a/-MoF variance	/e/-MoF variance	/u/-MoF variance

^a^MiF: mild-group filtering.

^b^MoF: moderate-group filtering.

### Data Analysis

#### Linear Discriminant Analysis Considering the Average and Variance Indices of the DTW Distance

The Mann-Whitney *U* test was used to determine whether there was any statistical significance between the mild and moderate I groups. This test was performed on 12 indices that measured the average and variance of the DTW distance for the 3 vowels /a/, /e/, and /u/. A significance level of 1% was established, with the null hypothesis of no statistical significance between the 2 groups. Box plots and linear discriminant analysis (LDA) were used to determine the indicators of the 3 vowels most effective for determining statistical significance between the 2 groups. The confusion matrix obtained from the LDA results was displayed with a specific index for the true positive rate (TPR), true negative rate (TNR), and balanced accuracy (BA). The boxplot function was calculated and plotted using the R *ggplot* package (version 3.4.0), and the LDA function was calculated using the R *MASS* package (version 7.3).

#### Generalized Linear Model With the DTW distance

The significant indices from the 4 categories of DTW distance were used to distinguish between severity levels (mild or moderate I). These indices were then used as explanatory variables to create generalized linear models (GLMs) for each vowel. A 5-fold cross-validation method with 110 waveform samples was used to train the model for each vowel, which was then used to predict the severity classification. R was used for GLM modeling and label prediction. The *pROC* package (version 1.18.0) for R was used to obtain the receiver operating characteristic (ROC) curve and calculate the area under the curve (AUC), whereas confusion matrices were generated using the *Caret* package (version 6.0) for R.

### Ethics Approval

The study was conducted in accordance with the Declaration of Helsinki and was approved by the Ethics Committee of Kanagawa University of Human Services (SHI 3-001, dated May 27, 2021, and SHI 26, dated November 25, 2021). Informed consent was obtained from all participants involved in the study.

## Results

### Participants

In June 2021 and March 2022, approximately 540,000 COVID-19 patients were recorded in Kanagawa prefecture. After requesting approximately 10,000 people to participate in the study, our study recruited 295 participants in the same period, of whom 291 were eligible to participate because participants who did not meet the inclusion criteria, such as minors, those with invalid data registration, or those who withdrew midway through evaluation, were excluded.

**Figure 3:**
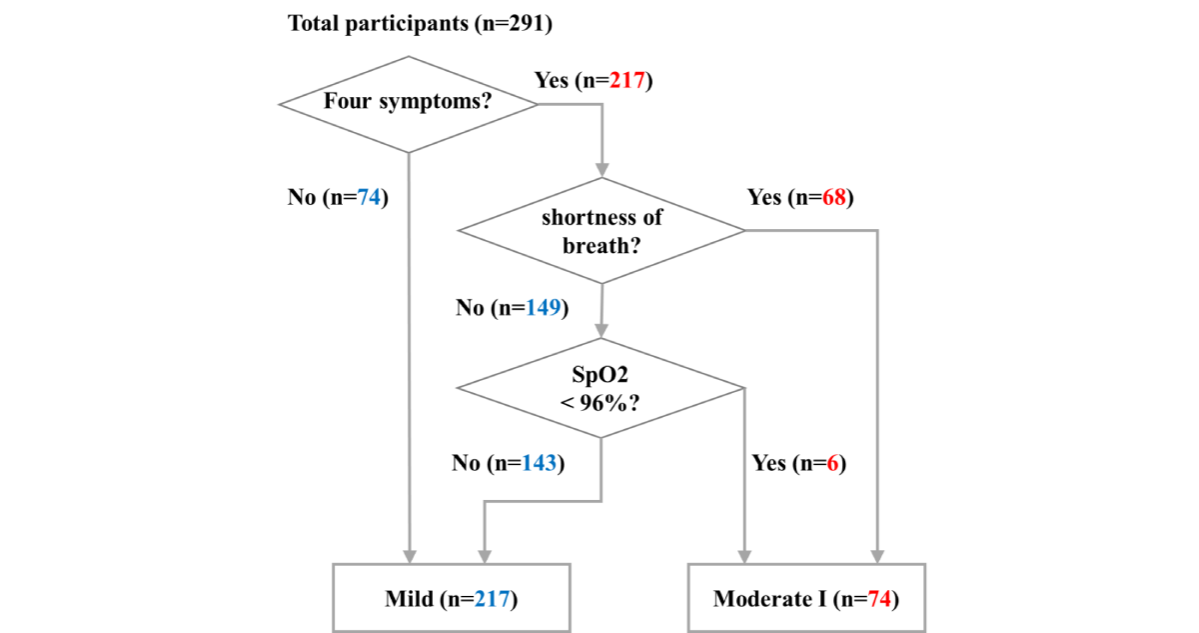
Flowchart showing classification of 291 participants into the mild illness and moderate illness I groups. “Four symptoms” refers to the 4 major symptoms: coughing, throat pain, chest pain, and shortness of breath. SpO2: oxygen saturation.

Seventy-four participants who reported no symptoms of coughing, throat pain, chest pain, or SoB during recuperation were assigned to the mild group. Of the 217 participants who reported any symptoms, 68 of them with symptoms of SoB were assigned to the moderate I group. Of the 149 participants who reported symptoms other than SoB, 6 of them with SpO_2_ values less than 96% were assigned to the moderate I group, and 143 participants who reported SpO_2_ values of no less than 96% were assigned to the mild group. The 291 participants were classified into 2 groups: 217 as mild and 74 as moderate I. Figure 3 shows a flowchart of study participation.

The primary periods of infection in Japan were during the Delta period, from July to December 2021, and the Omicron period, from January to June 2022. According to previous reports [16,17], COVID-19 exhibits varying levels of infectivity, severity, and symptoms, depending on the type of mutant strain present. We identified the time of infection in Japan and carefully matched the 291 study participants who had already been labeled into 2 groups. Table 4 shows the attribution matrix for the participants by the time of infection, sex, and severity. Table 5 shows the attribution matrix for the same sample based on the time of infection, sex, and age group. Finally, 110 participants (61 with mild illness and 49 with moderate illness I) were included in the study.

**Table 4:** Participants’ attribution matrix by the time of infection, sex, and severity (n=110).

Severity		Delta period^a^ (n=46), participants, n	Omicron period^b^ (n=64), participants, n
**Mild illness (n=** **61)**			
	Male	14	16
	Female	13	18
	Total	27	34
**Moderate illness I (n=49)**			
	Male	10	16
	Female	9	14
	Total	19	30

^a^Delta period: July 2021 to December 2021.

^b^Omicron period: January 2022 to June 2022.

**Table 5:** Participants’ attribution matrix by the time of infection, sex, and age group (n=110).

Age group (years)			Delta period^a^ (n=46), participants, n		Omicron period^b^ (n=64), participants, n
**20-29**					
	Male	9		10	
	Female	16		15	
	Total	25		25	
**30-39**					
	Male	4		9	
	Female	1		9	
	Total	5		18	
**40-49**					
	Male	7		6	
	Female	2		5	
	Total	9		11	
**50-59**					
	Male	3		4	
	Female	3		3	
	Total	6		7	
**60-69**					
	Male	0		2	
	Female	0		0	
	Total	0		2	
≥**70**					
	Male	1		1	
	Female	0		0	
	Total	1		1	

^a^Delta period: July 2021 to December 2021.

^b^Omicron period: January 2022 to June 2022.

### Linear Discriminant Analysis Considering the Average and Variance Indices of the DTW Distance

#### Distribution of the Average and Variance Indices of the DTW Distance

Table 6 displays the Mann-Whitney *U* test results for the 2 groups based on 3 vowels and 4 indicators. Of the 12 indices, 6 were found to be significant; they included /u/-MiF average, /a/-MiF variance, /e/-MiF variance, /a/-MoF variance, /e/-MoF variance, and /u/-MoF variance. The only index that was significant among the average indices was /u/-MiF average, while /u/-MiF variance was the only insignificant index among the variance indices. This indicates that the variance indices were more significant overall.

**Figure 4:**
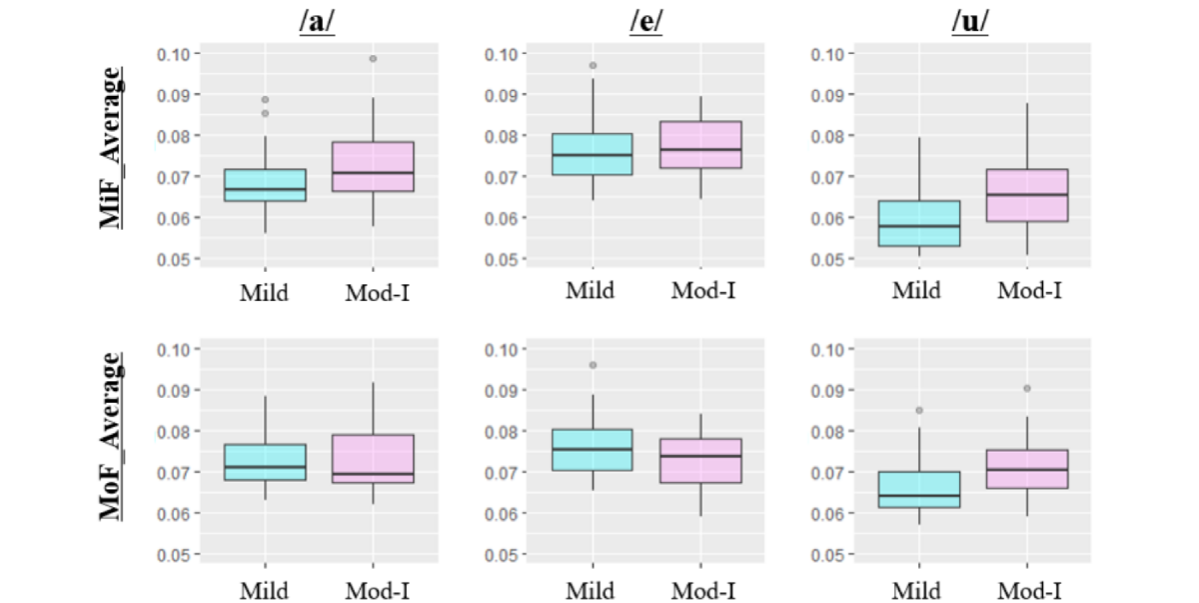
Distribution in the 2 groups of the average index for the dynamic time warping distance (MiF average and MoF average). MiF: mild-group filtering; MoF: moderate-group filtering.

**Figure 5:**
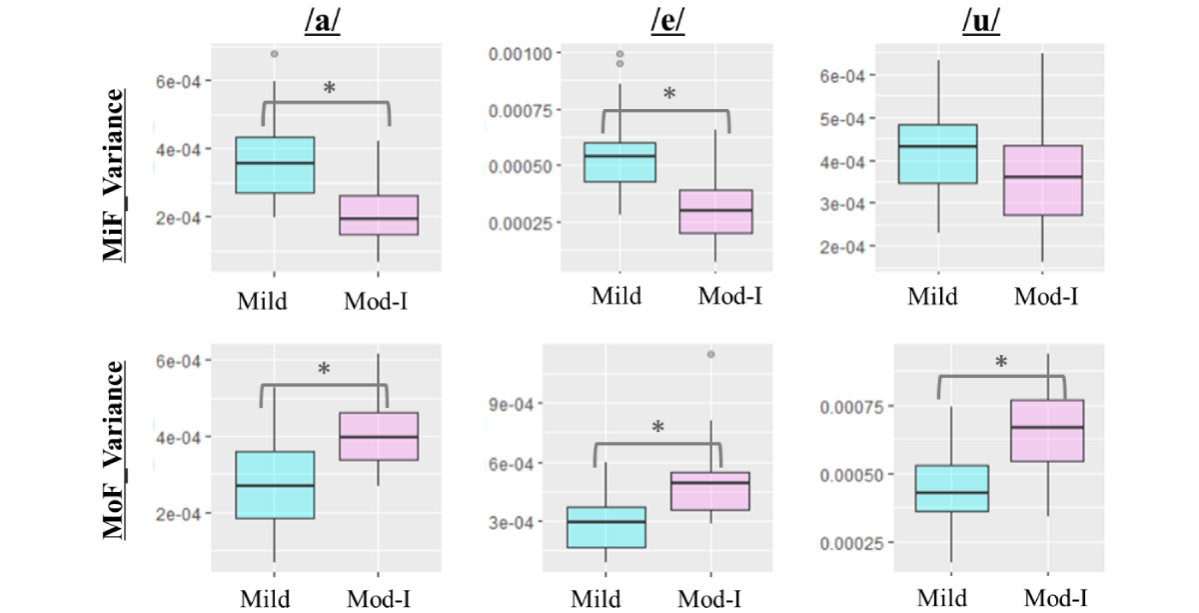
Distribution in the 2 groups of the variance indices for the dynamic time warping distance (MiF variance and MoF variance) *P<.001. MiF: mild-group filtering; MoF: moderate-group filtering.

Figures 4 and 5 illustrate the distributions of MiF average and MoF average, as well as MiF variance and MoF variance, for the 2 groups.

**Table 6:** Results of the Mann-Whitney U test for mild illness and moderate illness I group classification.

Indices	Vowels, *P* values		
	/a/	/e/	/u/
MiF^a^ average	.03	.71	<.001
MoF^b^ average	.60	.43	.03
MiF variance	<.001	<.001	.45
MoF variance	<.001	<.001	<.001

^a^MiF: mild-group filtering.

^b^MoF: moderate-group filtering.

#### Results for LDA

**Figure 6:**
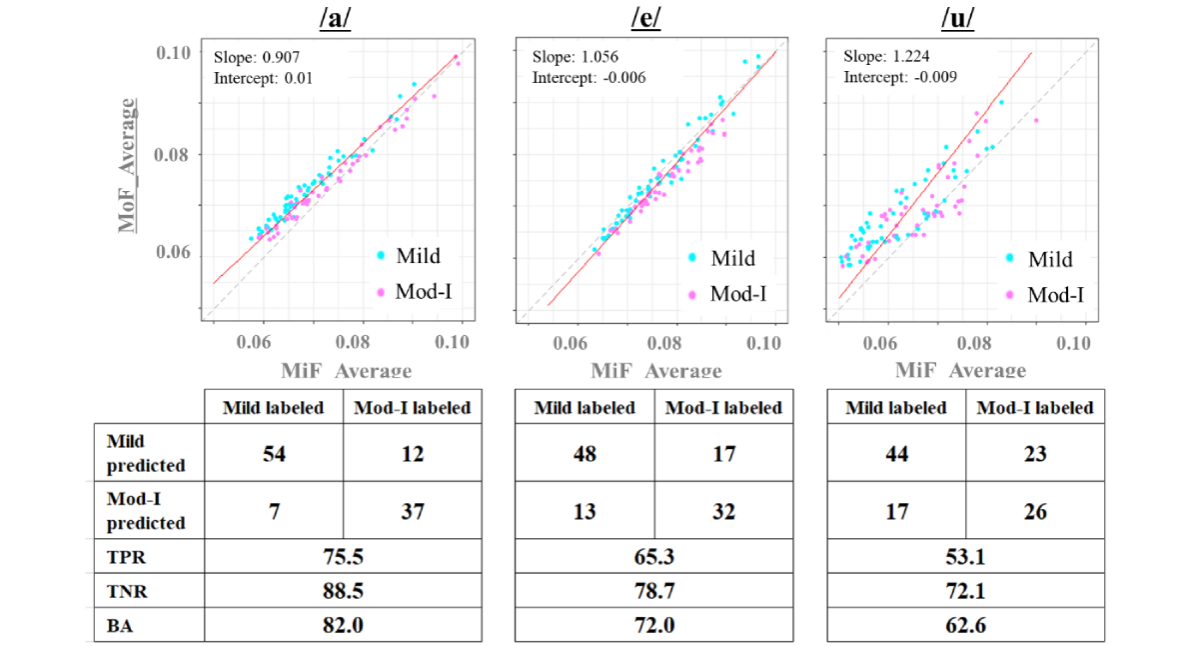
Linear discriminant analysis results and confusion matrix of the MiF average and MoF average indices. BA: balanced accuracy; MiF: mild-group filtering; MoF: moderate-group filtering; TPR: true positive rate; TNR: true negative rate.

**Figure 7:**
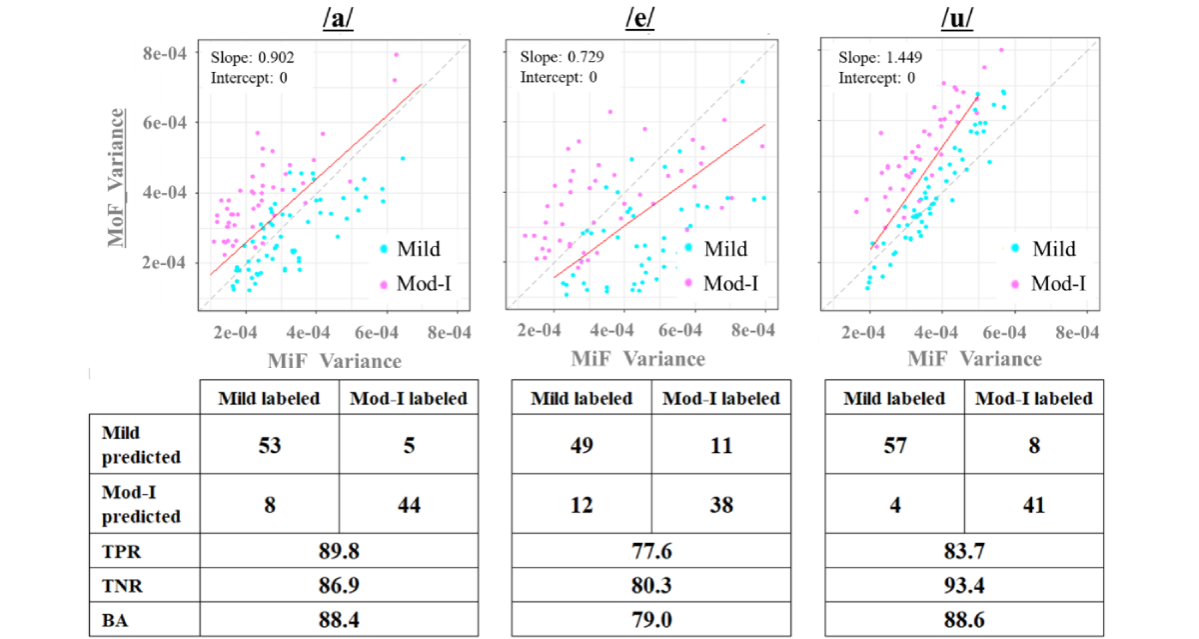
Linear discriminant analysis results and confusion matrix of the MiF-variance and MoF-variance indices. BA: balanced accuracy; MiF: mild-group filtering; MoF: moderate-group filtering; TPR: true positive rate; TNR: true negative rate.

Figure 6 shows a scatter plot of the average indices, and Figure 7 shows a scatter plot of the variance indices of the DTW distance, along with the confusion matrix, TPR, TNR, and BA values of the LDA. The straight line represents the discriminant line obtained using LDA. The variance indices of the DTW distance provided overall superior results for the classification indicators, including TPR, TNR, and BA, of the confusion matrix compared to the average indices. The ease of classification can be visually verified by observing the plots achieved via LDA.

#### GLM With the Variance Index of the DTW Distance

**Figure 8:**
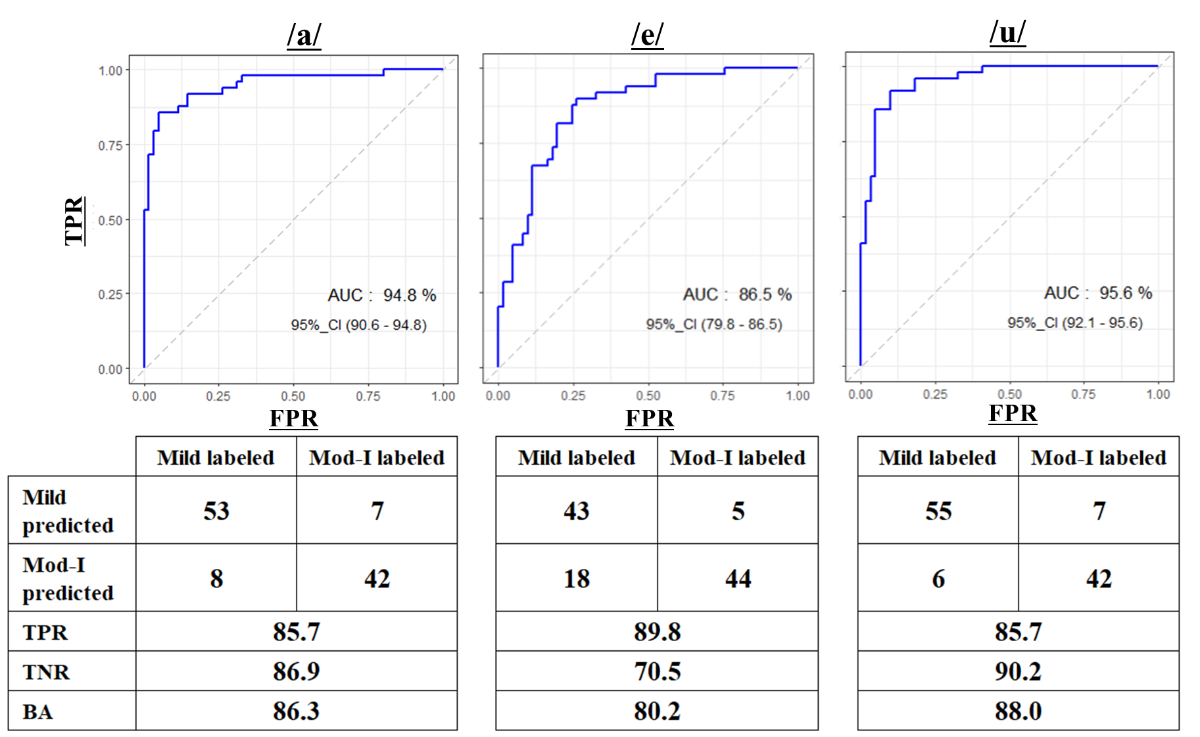
ROC/AUC results of the generalized linear model with confusion matrices for the mild-group filtering variance and moderate-group filtering variance indices. AUC: area under the curve; BA: balanced accuracy; FPR: false positive rate; TPR: true positive rate; TNR: true negative rate; ROC: receiver operating characteristics curve.

We used the variance indices of the DTW distance as predictors of the GLM model because they achieved better classification performances than the average indices. Figure 8 shows the ROC and AUC values of the GLM model for each vowel with the confusion matrix, including the TPR, TNR, and BA data. The models of all 3 vowels provided high model performance in terms of the AUC and mid-to-high accuracy for TPR, TNR, and BA.

## Discussion

### Principal Results

This feasibility study demonstrated that the DTW distance–based voice biomarkers generated by the GLM had a balanced accuracy ranging from 80.2% to 88% and high model performance, indicated by the AUC ranging from 86.5% to 96.5%, for 3 vowels when classifying between mild illness and moderate illness I in COVID-19 patients.

### Comparison With Prior Work

#### Key 1D Features of Acoustic Parameters

Sondhi et al [10] and Pah et al [18] found that the classification of subjects with and without COVID-19 was possible using jitter, shimmer, and HNR indices; however, they did not demonstrate classification using models incorporating these parameters. Pah et al [18] stated, “The statistical analysis and SVM classification indicated that the voice features of sustained phoneme corresponding to vocal tract modulation (Mel Frequency Cepstral Coefficient (MFCC), Formants, Vocal-tract Length, and Intensity-SD) could potentially be adopted as a COVID-19 biomarker compared to the features of vocal fold vibration (jitter, shimmer, pitch, HNR, and NHR)” [18]. This suggests that a simple model using only jitter, shimmer, and HNR is not able to differentiate between subjects with and without disease. Therefore, we believe that approaches such as machine learning and deep learning are essential for performing pathophysiological analyses such as classification, presence or absence determination, and monitoring using key 1D features of acoustic parameters, such as MFCC and formants.

#### 2D Feature Matching of DTW algorithms

In this study, the variance indices of the DTW distance were significantly different from the average indices. It appears that diseases have a wide-ranging impact on the voices of patients, making it challenging to assess and categorize voices at the desired level. To address this issue, machine learning–based voice analysis systems that focus on learning 1D parameters such as jitter and shimmer have been used, and tuning using single-dimensional key features such as spectral and prosodic speech features may be conducted [18-21]. Conversely, our 2D feature matching of the waveform using DTW algorithms may provide a more direct and practical method in the domain of binary classifications.

#### Advantages of the Standardization of Waveform Samples

By standardizing the time and power axes of the waveform samples in the DTW algorithm before computing, the fundamental frequency (F0) and volume were consequently transformed as parts of the elements forming a 10-cycle waveform in the unit envelope. Confounding factors derived from fundamental frequencies that vary by sex and age can be avoided as much as possible in advance, allowing the direct evaluation of the classification results by the DTW distance [8,19,22,23]. For this study, we examined 110 wavelet samples to determine the coefficient of variation (CV) of F0 estimates before and after standardization. Our goal was to analyze the variety of F0 distribution based on sex and age groups. Our results indicated that the CV of the wavelets without standardization ranged from 10.31% to 11.4% for the 3 long vowels /a/, /e/, and /u/, which is considered a significant confounding factor. However, after standardization, the CV ranged from 0.81% to 2.4%, which was significantly reduced and, therefore, effective in minimizing confounding factors. Multimedia Appendix 1 provides more details and comparison figures.

### Why Select a 10-Cycle Waveform as a Unit Sample?

Our previous pilot study using the DTW algorithm to differentiate between the voices of subjects with and without COVID-19 investigated the use of different waveforms with 1, 3, 5, 10, 20, 30, and 50 cycles. Among these waveform cycles, 10 and 20 cycles resulted in reasonable discrimination between the subjects with and without COVID-19 for all 3 subjects tested. (A summary of the test results is shown in Multimedia Appendix 2.) We selected a 10-cycle waveform, rather than a 20-cycle waveform, in order to reduce as much as possible the total computing cost incurred when using the DTW algorithm (see the section “Computing Cost”).

#### Robustness to Noise

While recent smartphones have improved recording features that can aid in voice analysis [24], it should be noted that the environmental noises present during the recording process were not completely controllable because the voice samples used in this study were self-recorded by the participants with their own smartphones. As Qi [25] reported, “DTW was evaluated using both synthetic and natural voices, and significant reductions in noise were achieved.” Because the DTW distance is considered to be robust to noise, it may be more practical when used for classification purposes with voices obtained from the real world compared to acoustic parameters such as jitter, shimmer, and HNR, which are considered to be sensitive to environmental noise [8,18].

#### Sample Size Consideration

There are known statistical correlations between significance level, power, effect size, and sample size [26]. We tested the validity of the sample size used in this study of 110 participants (61 in the mild group and 49 in the moderate I group). We calculated an effect size of 0.666 with the significance level of 1% used in this study and a power of 0.8, which generally meets requirements from a statistical point of view [27]. For the calculation, the *pwr* package (version 1.3.0) of R was used. A Cohen *d* score of 0.5 is regarded as a medium effect size, and 0.8 is regarded as a large effect size [28]. Therefore, we believe that the sample size used in this study was appropriate because the effect size of 0.666 is between medium and large. (These validation processes are disclosed in Multimedia Appendix 3.)

#### Future Expectations

DTW distance–based voice biomarkers may effectively supplement pulse oximeters as an objective indicator when distinguishing moderate illness I from mild illness among patients during recuperation. Even if pulse oximeters are scarce, this biomarker can be accessed through a patient’s smartphone. If persons with disease recuperating at home can detect a worsening of symptoms to moderate illness I based on changes in their voice, they will be able to determine whether they should seek medical care. In addition, this system may allow health care providers to use voice biomarkers in addition to body temperature and pulse oximeter readings as objective and quantitative indicators to properly diagnose worsening symptoms and expedite inpatient treatment.

### Limitations

#### Computing Cost

In this study, our approach involved standardizing waveform samples, comprehensively computing the DTW distance for each sample, and subsequently using the resulting indices to determine the severity of illness using a GLM. However, this approach is considered computationally expensive, making it unsuitable for integration into standalone smartphone apps. Nevertheless, it can be used in the cloud. Despite current limitations, significant advancements in network transmission speed and information technology suggest that it may soon be practically applicable. Another potential solution for reducing computation time is to measure the DTW distance from a greater variety of representative samples as the number of cases increases. However, this remains a topic for future study.

#### Patient Bias

Although the definition of moderate illness I that was used in this study is based on pulse oximeter readings (SpO_2_ 93%-96%) or subjective reports from patients of their clinical condition (ie, SoB, as shown in Table 1), errors during labeling of the voice samples due to patient bias may have occurred as different individuals may have varying methods of describing the sensation of SoB. Unfortunately, this issue is difficult to overcome considering the use of patient-reported data. However, the inclusion of objective indicators such as voice biomarkers during the diagnosis-making process may allow for more objective labeling of data in the future.

### Conclusions

Medical treatments for COVID-19 vary depending on the severity of the illness. Patients with mild illness may need only to recuperate at home or a designated facility, whereas patients with moderate illness I may need to be hospitalized. In this study, the DTW distance–based voice biomarker was tested for distinguishing between mild and moderate illness I. A balanced accuracy ranging from 80.2% to 88% was achieved, and the model performance indicated by the AUC ranged from 86.5% to 96.5% for the vowels /a/, /e/, and /u/. This voice biomarker system can be used in case of an unexpected shortage of pulse oximeters as an alternative and cost-effective method for monitoring worsening medical conditions in patients with mild illness that are recuperating at home or a medical facility.

## Appendix

Multimedia Appendix 1 Standardization and confounding.

Multimedia Appendix 2 Validation for waveform cycles.

Multimedia Appendix 3 Sample size consideration.

## Abbreviations

AUC: area under the curve
BA: balanced accuracy
EPV10: events per variable 10
DTW: dynamic time warping
GLM: generalized linear model
HNR: harmonic-to-noise ratio
LDA: linear discriminant analysis
MiF: mild-group filtering
MFCC: mel-frequency cepstral coefficient
MoF: moderate-group filtering
ROC: receiving operator curve
SoB: shortness of breath
SpO2: oxygen saturation
TPR: true positive rate
TNR: true negative rate

## Data Availability

The data sets generated during and/or analyzed during this study are not publicly available due to privacy and ethical restrictions but are available from the corresponding author on reasonable request.
