# Supplementary material for: Severity Classification Using Dynamic Time Warping–Based Voice Biomarkers for Patients With COVID-19: Feasibility Cross-Sectional Study
Source: JMIR Biomed Eng. 2023 Nov 6;8:e50924. doi: 10.2196/50924 (PMC10631492; doi:10.2196/50924)

Multimedia Appendix 1: (1) Standardization to Power and Time Axes

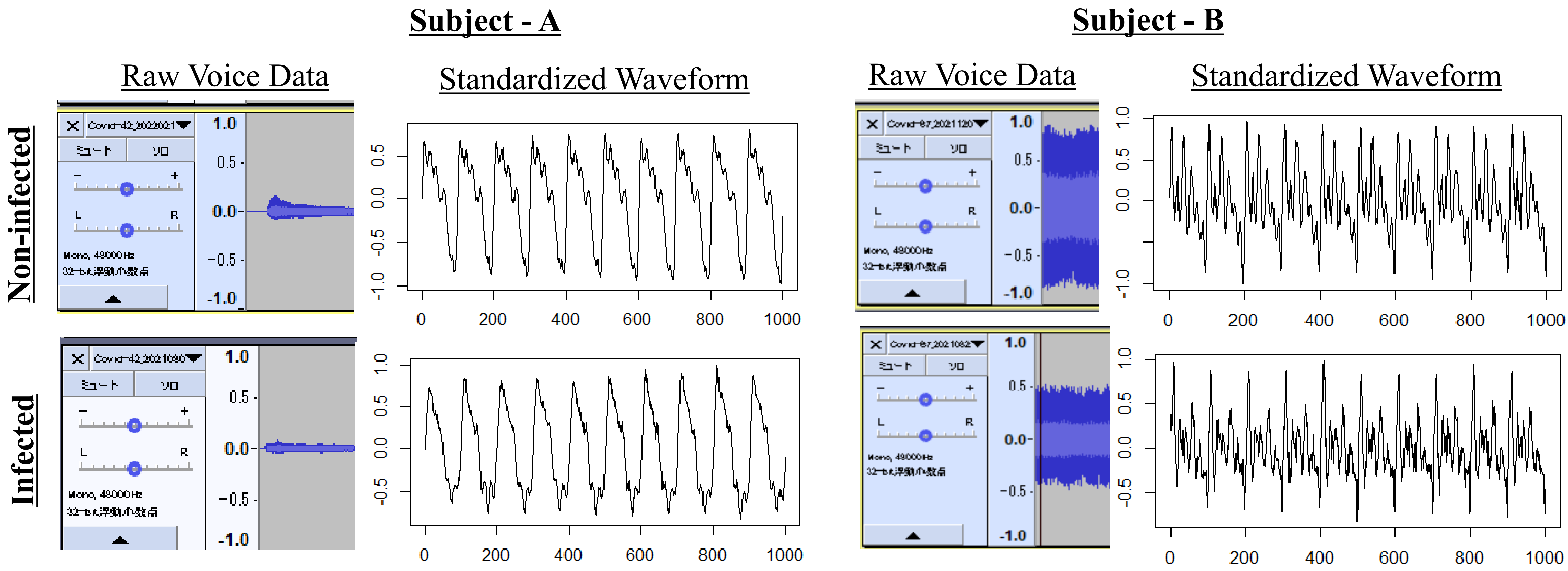

- All unit waveform samples have fitted within the *envelope,* resulting from standardization to power and time axis.
- This contributed to better feature-matching because the confounding factors of fundamental frequency (F0) and vocal volume among the participants were canceled.

Multimedia Appendix 1: (2) Additional Analysis for F0 Confounding

The fundamental frequency (F0) is known to vary with gender and age. However, when comparing wavelet features using the DTW algorithm, the difference in F0 is considered a confounding factor that makes disease severity misleading.

It is, therefore, necessary to standardize (or normalize) each wavelet to the time axis to remove a confounding factor before calculating the DTW distance.

The figures in the next slides indicate the variety of F0 distribution by sex and age groups. It also shows the effectiveness of the normalization on the wavelets that were used in this study.

110 Wavelets' F0 Distribution: Not Normalized (left) and Normalized (right)

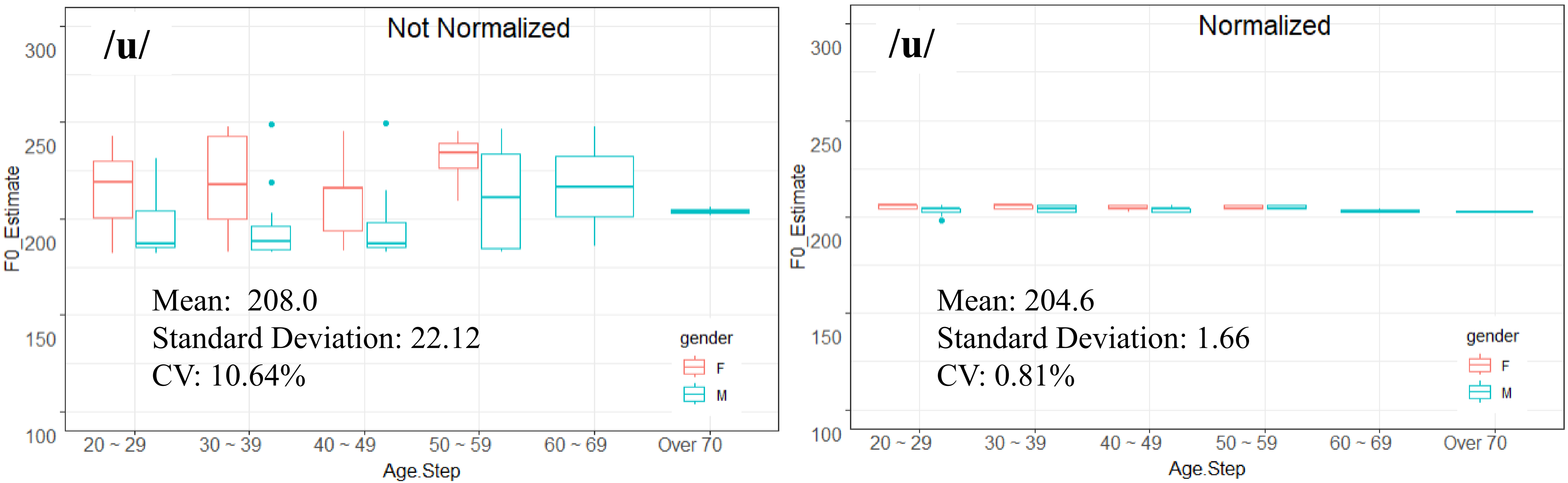

F0 was estimated using the Fast Fourier Transform function with additional sorting programming in R. 110 samples of 10-cycle wavelets were subjected to F0 estimation in either state, "not-normalized," or "normalize." The result of the /u/ vowel is shown here with the Coefficient of Variance(CV), 10.64%(not normalized) to 0.81%(normalized). /a/ and /e/ are shown in the next slide.

110 Wavelets' F0 Distribution: Not Normalized (left) and Normalized (right)

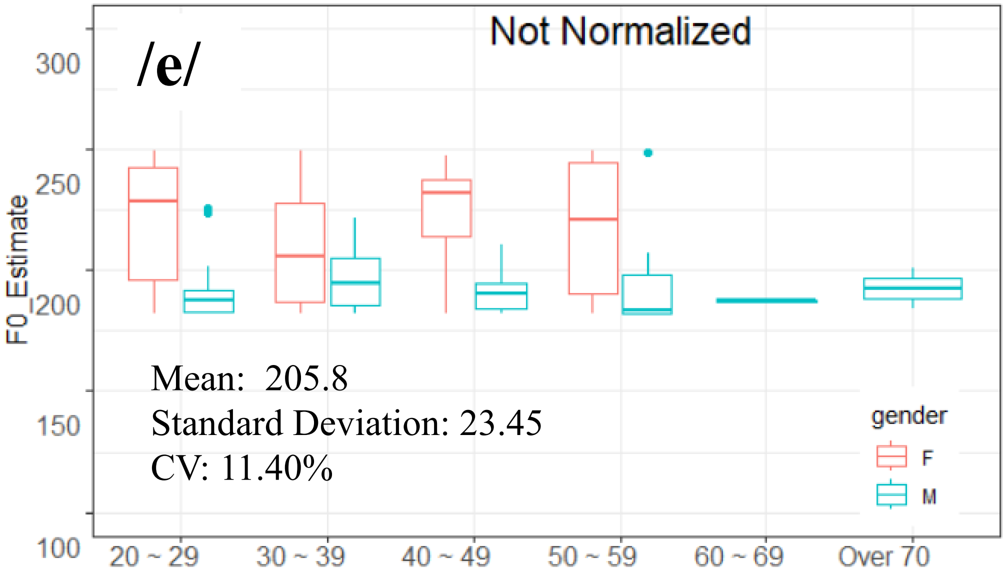

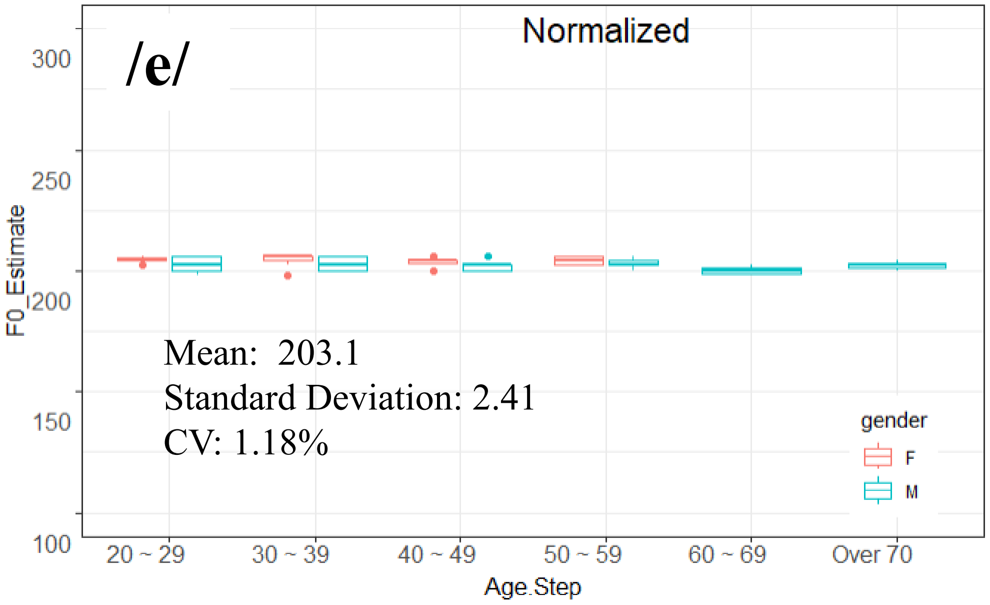

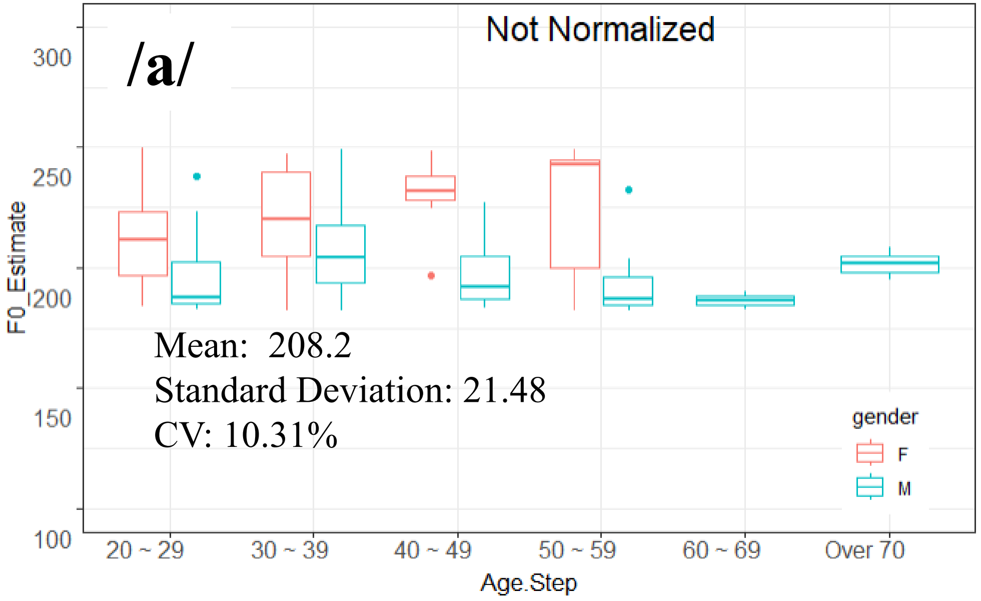

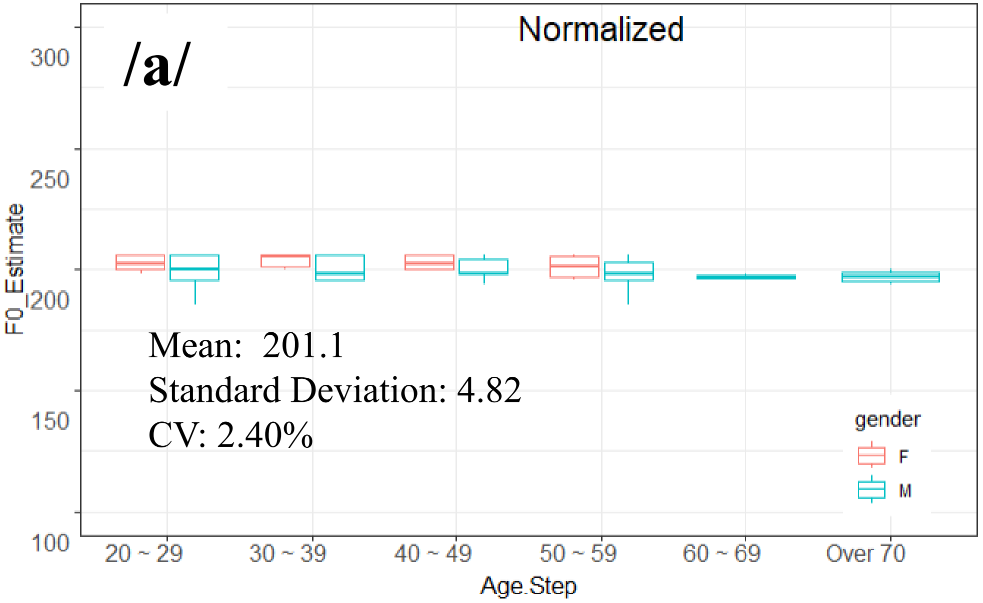

Supplement: Multimedia Appendix 1 [file biomedeng_v8i1e50924_app1.pdf]
