# Supplementary material for: Severity Classification Using Dynamic Time Warping–Based Voice Biomarkers for Patients With COVID-19: Feasibility Cross-Sectional Study
Source: JMIR Biomed Eng. 2023 Nov 6;8:e50924. doi: 10.2196/50924 (PMC10631492; doi:10.2196/50924)
Supplement: Multimedia Appendix 2 [file biomedeng_v8i1e50924_app2.pdf]

Multimedia Appendix 2: Validation for Waveform Cycles

- A pilot study was executed to validate the appropriate waveform cycles using the following samples.

- Samples of 1-, 3-, 5-, 10-, 20-, 30- and 50-cycle waveforms of “infected” and “non-infected” subjects

- The DTW algorithm calculated interpersonal voice classification per waveform cycle and plotted to determine the most adequate waveform cycle for the study.

Samples of 1-, 3-, 5, and 10-cycle waveforms from the same voice sample

Infected Waveform | Non-Infected Waveform

1-cycle

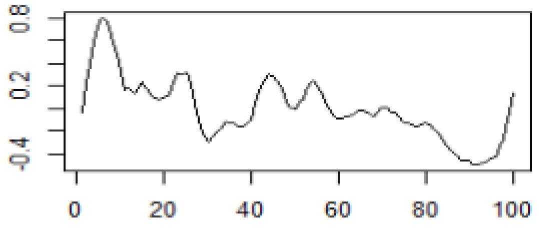

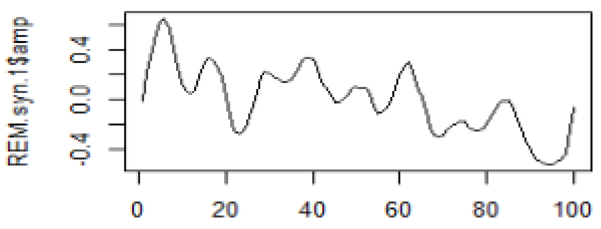

3-cycle

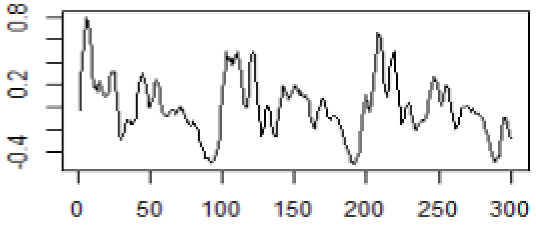

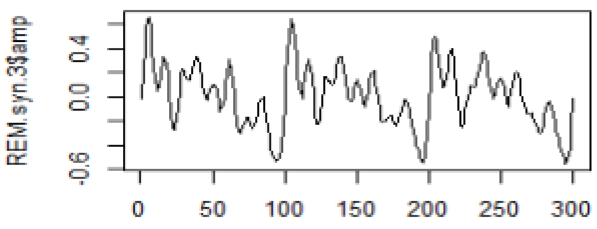

5-cycle

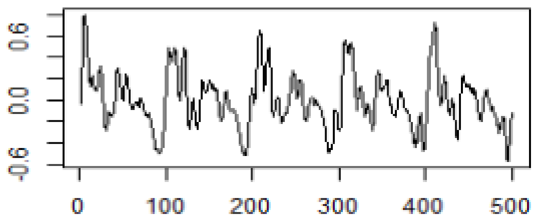

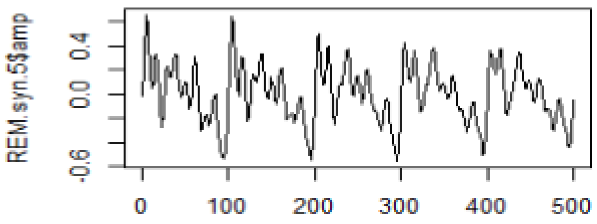

10-cycle

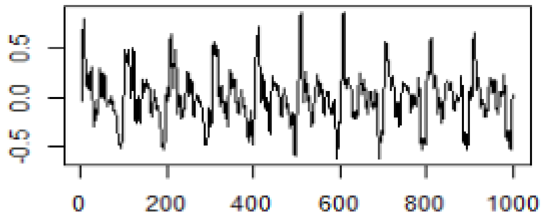

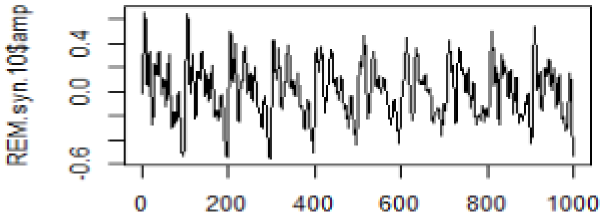

Samples of 20-, 30-, 50-cycle waveforms from the same voice sample

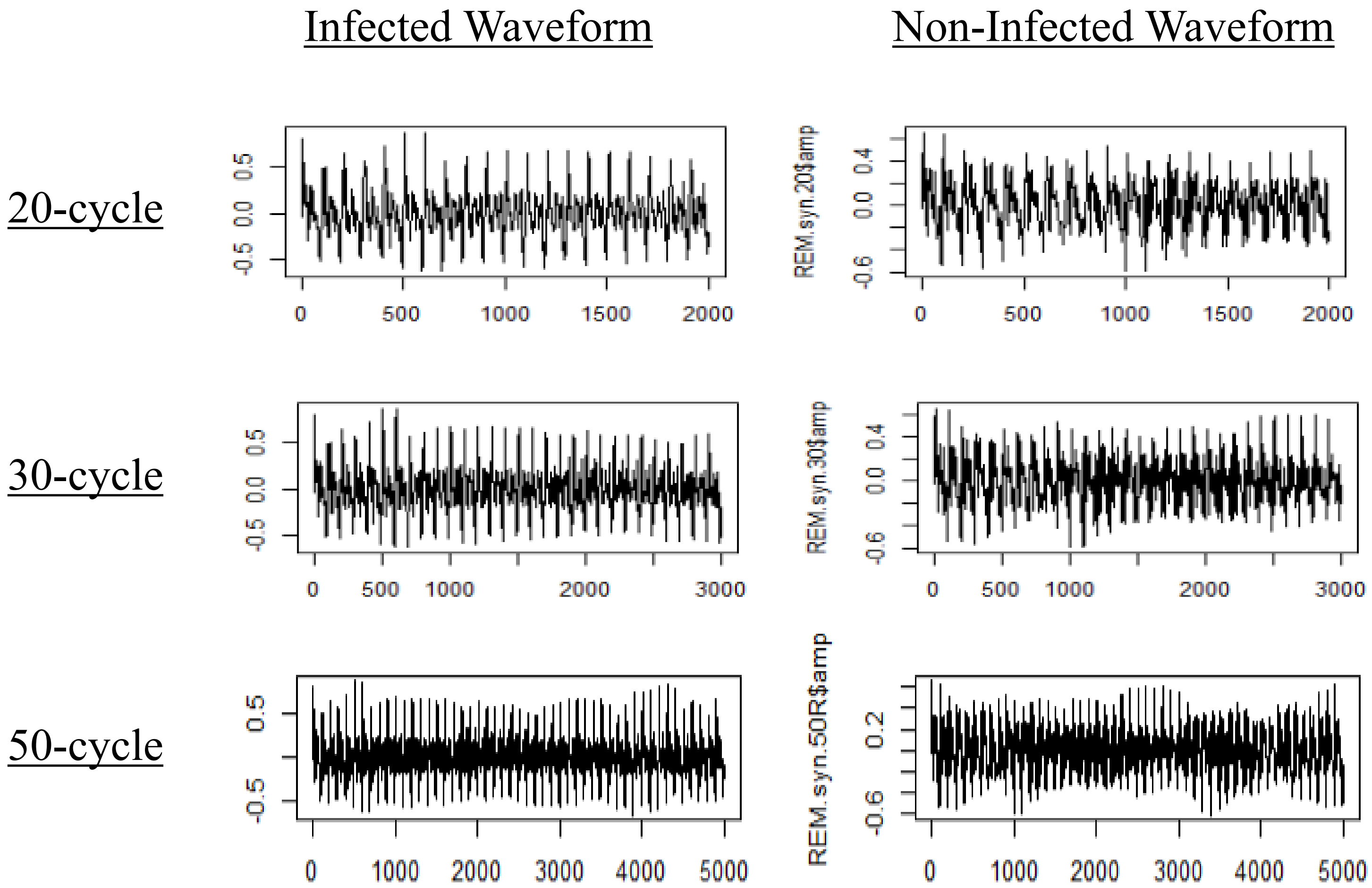

Interpersonal Voice Classification per Waveform Cycle

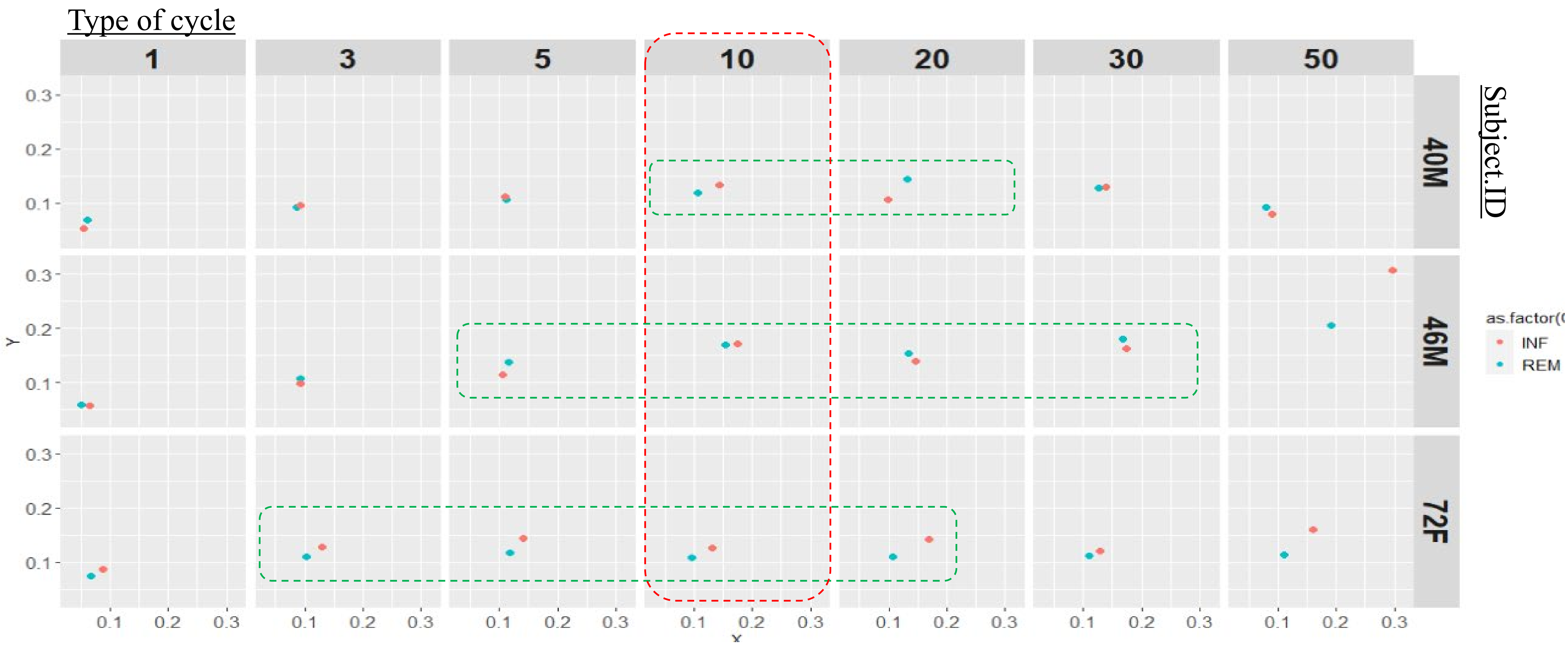

- The distance between the two points “infected” (INF, red dots) and “in remission” (REM, blue dots) seems to be adequate for discrimination in the range of 3 to 30 cycles in the three test cases (40M, 46M, 72F). Among them, waveforms with 10 or 20 cycles were commonly discriminable distances for all three subjects. In this study, the 10-cycle waveform was selected as the unit sample waveform rather than the 20-cycle due to computational cost.
