# Supplementary material for: Severity Classification Using Dynamic Time Warping–Based Voice Biomarkers for Patients With COVID-19: Feasibility Cross-Sectional Study
Source: JMIR Biomed Eng. 2023 Nov 6;8:e50924. doi: 10.2196/50924 (PMC10631492; doi:10.2196/50924)
Supplement: Multimedia Appendix 3 [file biomedeng_v8i1e50924_app3.pdf]

Multimedia Appendix 3: Sample Size Consideration

```{r}
pwr.t.test(d = 0.5, power = 0.8, sig.level = 0.01)

pwr.t.test(d = 0.8, power = 0.8, sig.level = 0.01)

pwr.t2n.test(n1 = 49, n2 = 61, power = 0.8, sig.level = 0.01)
```

```
     Two-sample t test power calculation

              n = 95.10364
              d = 0.5
      sig.level = 0.01
          power = 0.8
    alternative = two.sided

NOTE: n is number in *each* group


     Two-sample t test power calculation

              n = 38.18831
              d = 0.8
      sig.level = 0.01
          power = 0.8
    alternative = two.sided

NOTE: n is number in *each* group


     t test power calculation

             n1 = 49
             n2 = 61
              d = 0.6658522
      sig.level = 0.01
          power = 0.8
    alternative = two.sided
```

- At a significance level of 1% and a power of 0.8, each group sample size was calculated for the range of Cohen's effect size from 0.5 (medium effect) to 0.8 (large effect).
- The range was from 39 each (effect size: 0.8) to 96 each (effect size: 0.5). Each of our sample sizes (49 for moderate I and 61 for mild) is within this range.
- Then we calculated Cohen's effect size using different sample sizes of two groups and found it was about 0.666, which was considered effective to classify two groups as far as p-values are less than 1%.
- We concluded that the sample size we used for this feasibility study was validated.

Cohen_d Calculation

p-value	Ah	Eh	Uh
MiF_average	0.034	0.7115	7.66E-04
MoF_average	0.6029	0.4326	0.0316
MiF_variance	2.09E-05	8.28E-05	0.4468
MoF_variance	3.95E-05	5.81E-05	2.93E-06

Cohen_d	Ah	Eh	Uh
MiF_average	0.41	0.07	0.67
MoF_average	0.15	0.31	0.37
MiF_variance	0.74	0.78	0.14
MoF_variance	0.91	0.8	0.97

- We calculated Cohen's effect size (bottom left) for each index using the P-values shown in Table 6 of the manuscript (top left)
- The effect sizes for the *variance* indices used for GLM classification were in the high range of 0.74 to 0.97. (except for 0.14 for the Uh-MiF-variance)
